# Focal impaired awareness seizures in a rodent model: A functional anatomy

**DOI:** 10.1002/epi4.12563

**Published:** 2021-12-17

**Authors:** Nadia Adotevi, Jaideep Kapur

**Affiliations:** ^1^ Department of Neurology University of Virginia Charlottesville Virginia USA; ^2^ UVA Brain Institute University of Virginia Charlottesville Virginia USA

**Keywords:** c‐Fos, comorbidities, depression, hippocampus, kindling, memory, TRAP

## Abstract

**Objective:**

Patients with temporal lobe epilepsy (TLE) frequently report debilitating comorbidities such as memory impairments, anxiety, and depression. An extensive neuronal network generates epileptic seizures and associated comorbidities, but a detailed description of this network is unavailable, which requires the generation of neuronal activation maps in experimental animals.

**Methods:**

We recorded electrographic seizures from the hippocampi during a kindling‐evoked focal impaired awareness seizure with observed freezing, facial twitching, and involuntary head bobbing. We mapped seizure circuits activated during these seizures by permanently tagging neurons through activity‐induced immediate early genes, combined with immunohistochemical approaches.

**Results:**

There was bilateral activation of circuits necessary for memory consolidation, including the hippocampal complex, entorhinal cortex, cingulate gyrus, retrosplenial cortex, piriform cortex, and septohippocampal complex in kindled animals compared with unstimulated awake behaving mice. Neuronal circuits in the ventral hippocampus, amygdala, and anterior cingulate cortex, which regulate the stress response of hypothalamic‐pituitary‐adrenal axis, were also markedly activated during a focal impaired awareness seizure.

**Significance:**

This study highlights neuronal circuits preferentially activated during a focal awareness impaired seizure in a rodent model. Many of the seizure‐activated neuronal circuits are critical modulators of memory consolidation and long‐term stress/depression response. The hijack of these memory and depression regulatory systems by a focal seizure could account for the frequent reports of comorbidities such as memory impairment and depression in many TLE patients.

**Key Points**

- We provide a detailed description of the extensive neuronal network that generates epileptic seizures and associated comorbidities
- Neuronal circuits activated during focal impaired awareness seizures are key modulators of memory consolidation and stress/depression response
- Seizure‐mediated hijack of memory and depression systems could account for frequent reports of memory difficulties and depression in many temporal lobe epilepsy (TLE) patients

## 1 INTRODUCTION

Temporal lobe epilepsy (TLE) is the most common form of epilepsy. In TLE, focal impaired awareness seizures commonly begin at a focus and spread by engaging a neuronal network with multiple nodes. Hippocampus is a common site of seizure origin in patients with TLE, and focal resection or ablation of the hippocampus can ameliorate seizures. However, resective surgery is not always successful because the remaining nodes of the TLE network may generate seizures.^1^ Interictal and ictal discharges impacting these extrahippocampal network nodes may be responsible for comorbidities associated with TLE, including memory deficits and anxiety and depression. Previous studies in patients using scalp and intracranial recordings, MRI, and fMRI allude to this. These studies provide valuable insights into the seizure network but lack spatial resolution at the cellular level, which requires the generation of neuronal activation maps in experimental animals.

Neuronal activation maps are integral to understanding the generation and propagation of seizures. They can define the structures and pathways involved in the progression of an ictal event from onset to termination. These maps are constructed with various metabolic markers that rapidly respond to synaptic and membrane electrical activity, including activity‐dependent immediate early genes (IEGs) such as c‐Fos. Activation of c‐Fos is a measure of neuron firing rate, depolarization, or expression of intracellular second messengers, occurring transiently in response to calcium influx.^2^ While c‐Fos provides cellular‐level labeling of neurons activated by a given stimulus, it has a poor temporal resolution and variable signal‐to‐noise ratio, primarily influenced by immunolabeling protocol and quality of antibodies. Transgenic mice, which express fluorescent reporters downstream of activity‐dependent promoters, offer much higher spatial and temporal resolution.^3^, ^4^, ^5^ These provide permanent tags to IEGs transiently activated by neuronal activity, based on tamoxifen‐dependent Cre recombinases.

We examined widespread activation patterns of a behavioral grade 2 seizure generated via electrical kindling of the hippocampus using activity reporter TRAP2 mice, which exhibit permanent expression of Cre‐driven tdTomato (TdT) in activated neurons in response to 4‐hydroxytamoxifen (4‐OHT) administration.^3^, ^5^ Kindling is an animal model of focal seizures initiated at a specific site. Applying repeated, discrete low‐intensity electrical current stimuli induce a progressive and permanent increase in seizure activity, with the initial focal seizures progressively evolving into convulsive events.^6^ These kindled animals exhibited behaviors including freezing, similar to observations during human focal impaired awareness seizures. However, we did not test awareness in experimental animals. Comparing these animals with awake behaving mice highlights the brain regions preferentially activated during a focal hippocampal seizure.

## 2 METHODS

### 2.1 Animals

The University of Virginia Animal Care and Use Committee approved all experimental protocols. We crossed mice‐expressing Cre‐ER under the regulation of the c‐Fos promoter (Fos^2A‐iCreER^; Jackson Laboratories, 030 323) with mice‐expressing TdT from the Rosa locus (B6. Cg‐Gt(ROSA)26Sor^tm9(CAG‐tdTomato)Hze/^J; Jackson Laboratories, 007 909) to generate TRAP2 mice. Genotypes were confirmed with tail genomic DNA using a kit from KAPA Biosystems. We used 7‐ to 12‐week‐old mice of both sexes and maintained them on a 12‐h light/dark cycle with ad libitum access to food and water.

### 2.2 Electrical kindling and video‐EEG monitoring

Mice were stereotaxically implanted with a stimulating bipolar electrode (constructed from two twisted insulated stainless steel wires) in the left ventral CA1 hippocampus, bilateral cortical electrodes, and a cerebellar reference electrode. They were rested for a week and then connected to a video‐EEG monitoring system as previously described.^7^ We determined an after‐discharge threshold (ADT) for each animal using a 1‐ms biphasic squared wave pulse at 60 Hz applied for 1 second to the bipolar hippocampal electrode. The initial stimulation was set at a pulse amplitude of 20 μA. Successive stimuli comprising 20 μA increases were applied at ~2‐minute intervals until a seizure with a duration of at least 10 seconds was observed for both cortical electrodes on the EEG. Afterward, electrical stimulation was performed daily with a pulse amplitude of 125% ADT. We scored seizures behaviorally using a modified Racine Scale, with freezing, facial twitching, and involuntary head bobbing classified as a grade 2/focal impaired awareness seizure. On average, animals exhibited a behavioral grade 2 seizure after 12 stimulations (12.5 ± 0.38; n = 46), similar in male and female animals. A control group of animals were similarly implanted and connected to the video‐EEG monitoring system but did not receive a stimulus. Animals were administered with a single injection of 4‐OHT (50 mg/kg; Sigma, H7904) within 90 minutes following a third grade 2 seizure with EEG manifestations. Matched electrode implanted, but unkindled control mice also received an injection at this time. We perfused the mice five or more days following injection to enable maximal TdT expression.

### 2.3 Immunohistochemistry

We cleared the tissue as previously described.^8^, ^9^ We perfused animals transcardially with a fixative solution consisting of 4% paraformaldehyde (PFA) and 4% acrylamide in 0.1 mol/L phosphate buffer, and brains postfixed overnight at 4°C. The slices with the thickness of 200 μm were sectioned on a vibratome (Leica VT1200S) into a solution of 1% acrylamide and 0.25% VA044 (Wako, NC0632395) in phosphate‐buffered saline (PBS), with overnight incubation at 4°C. Sections underwent degassing and a 2‐hour polymerization at 37°C, followed by clearing in 8% SDS in PBS until they were transparent.

Additionally, a cohort of animals was perfused transcardially with 4% PFA in 0.1 mol/L phosphate buffer. The brains were postfixed overnight at 4°C and then cryoprotected in 30% sucrose in PBS solution until sunken. Next, the brains were rapidly frozen in isobutane and sectioned on a cryostat (Leica CM1900) into 40‐μm‐thick slices. Following blocking of tissue sections (50 μL/mL of NGS and 10 mg/mL of BSA in PBS with 0.1% Triton X‐100 (PBST) overnight) to prevent nonspecific antibody binding, we immunolabeled sections using the following antibodies: mouse anti‐NeuN (1:500; Millipore, MAB377), rabbit anti‐ChAT (1:500, Invitrogen, PA5‐29653), Alexa Fluor 488 goat anti‐mouse (1:500; Invitrogen, A11029), and Alexa Fluor 488 goat anti‐rabbit (1:500; Invitrogen, A11008).

### 2.4 Image acquisition and analysis

Images were acquired with a Nikon C2 at 10X magnification (0.45 NA/0.17 WD) as Z‐stacks (200‐μm slices: 10‐μm Z‐distance, and 40‐μm slices: 5‐μm Z‐distance between consecutive images) using NIS‐Elements (Nikon Inc, AR 5.20.02). Channel configurations were set as 488‐nm FITC (NeuN, ChAT) and 561‐nm TRITC (TdT), with channel cross‐over excluded through band‐filtering sequential scanning. We used the same acquisition parameters to obtain images for both kindled and control unkindled mice.

Composite images were analyzed using Imaris software 9.7.2 (Bitplane Scientific). Accurate placement of the hippocampal stimulating electrode in the left ventral CA1 was verified in imaged sections. TdT and NeuN immunoreactivity represent neuronal activity and neuronal density, respectively. A region of interest encompassing each sampled brain region was marked, and cells were identified and counted using the automated spots module to determine the number of TdT^+^ neurons. At least three sections were analyzed per animal.

We prepared images for figures using Adobe Photoshop, with original images cropped and optimized for brightness and contrast. We defined outlines of brain nuclei and locations of labeled neurons based on the Franklin and Paxinos mouse brain atlas.^10^

### 2.5 Statistics

Statistical tests were performed using GraphPad Prism 9.0 (GraphPad Software). We tested normality using the Shapiro‐Wilk test and compared animal groups using an unpaired *t*‐test with Welch's correction. In figure legends, we present data as mean ± SEM with statistical significance set at *P* =  .05, and in figures, we display box‐and‐whisker plots where whiskers show the 5th through 95th percentiles.

## 3 RESULTS

### 3.1 Activation of the hippocampal complex and parahippocampal regions

**FIGURE 1:**
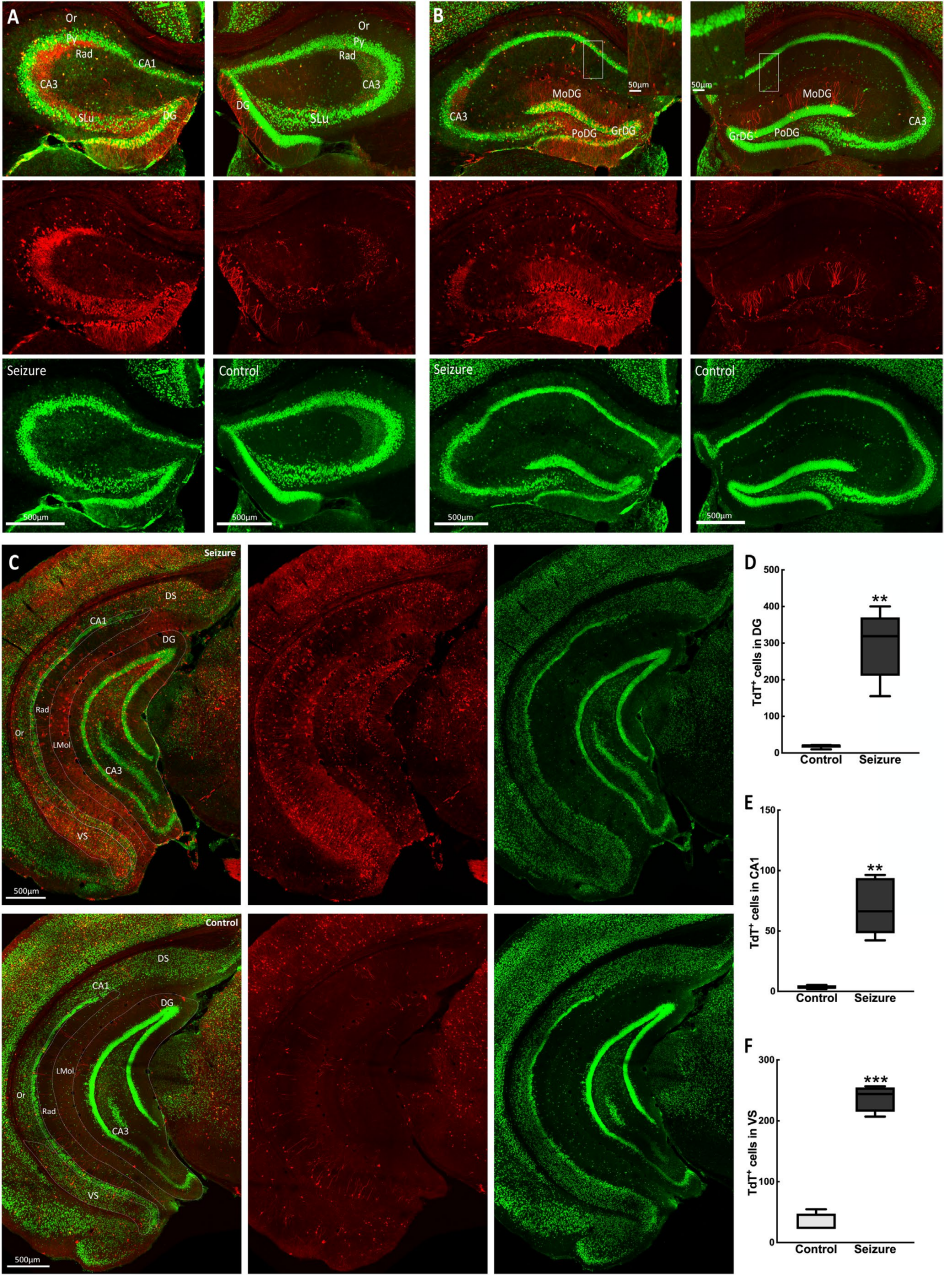
Neuronal activation in the hippocampus during a focal impaired awareness seizure. (A) Coronal images at bregma −1.23 mm showing TdT‐labeled cells only (red) and NeuN‐labeled neurons only (green) in the hippocampus of a mouse with a grade 2 behavioral seizure (left panel) and a control‐unstimulated mouse (right panel). (B) Images at bregma −1.67 mm showing TRAPed hippocampal neurons during a seizure (left panel) and in a control‐unstimulated mouse (right panel). Inserts in top panels show TdT‐labeled cells in the CA1 pyramidal cell layer. (C) Coronal images at bregma −3.39 mm showing TdT‐labeled neurons in the vental hippocampus of a mouse with a seizure (top panel) and a control mouse (bottom panel). Merged images of TdT‐ and NeuN‐labeled neurons are shown in the topmost panels in A and B, and in the leftmost panels in (C). (D) More TdT^+^ DGCs were activated in mice with a grade 2 seizure (control 18 ± 2, seizure 296 ± 41; n = 5 mice per group, *t*(4.02) = 6.72, *P* = .002). (E) Labeled dorsal CA1 pyramidal cells were more in mice with a grade 2 seizure (control 3 ± 1, seizure 70 ± 10; n = 5 mice per group, *t*(4.03) = 6.35, *P* = .003). (F) TdT^+^ cell activation was greater in the VS during a seizure (control 31 ± 8, seizure 237 ± 11; n = 4 mice per group, *t*(5.42) = 15.39, *P* = .001). Regions marked are DS, dorsal subiculum; GrDG, dentate granule cell layer; LMol, lacunosum moleculare layer; MoDG, dentate molecular layer; Or, oriens layer; PoDG, dentate polymorph layer/hilus; Py, pyramidal cell layer; Rad, radiatum layer; SLu, stratum lucidum; VS, ventral subiculum

Our seizure focus was in the hippocampus, so we first examined neuronal activation in the hippocampus and closely anatomically connected parahippocampal areas. Many more TdT^+^ neurons were present in the dentate gyrus (DG), CA3, CA1, and subiculum regions of the hippocampal complex (Figure 1) in a mouse with a behavioral grade 2 seizure compared with a control mouse. This higher activation was present in both the stimulated left and contralateral right hippocampi. The densely packed dentate granule cell layer (DGC) was prominently active, and TdT^+^ neurons were also present in the hilus. In contrast, we observed very few activated DGCs in unstimulated mice, which aligns with reports that there is sparse activation of DGCs at the resting state.^11^ The molecular layer of the DG is largely cell‐free, so we presume that labeling in this area was primarily granule cell dendrites and fibers of the perforant path and other extrinsic inputs (Figure 1B). In area CA3, pyramidal cells, stratum lucidum containing DGC mossy fiber terminals, and cells of the stratum oriens were labeled with TdTomato.

In CA1, there were labeled TdT^+^ pyramidal cells. This layer receives Schaffer collaterals from the CA3 subfield positive for TdT. These were present in both dorsal and ventral CA1 (Figure 1). We observed TRAPed neurons, and presumably glial cells in the subiculum, a major output structure of the hippocampus, perforated by axonal projections from the entorhinal cortex (EC) to DG. However, there was much sparser labeling in the proximal dorsal area bordering the CA1 compared with the ventral subiculum bordering the presubiculum (Figure 1C).

**FIGURE 2:**
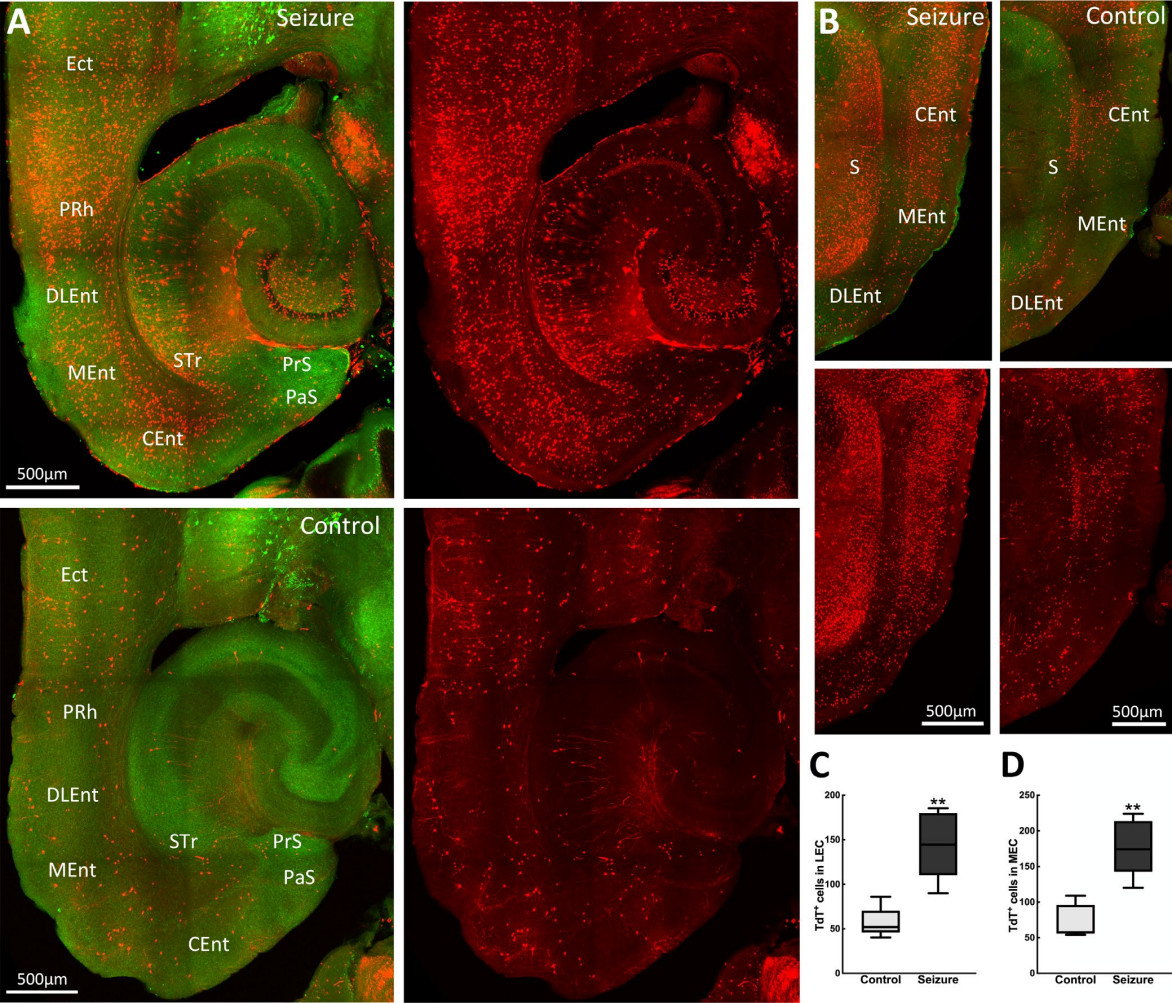
Parahippocampal structures were also activated. (A) Horizontal images at bregma −3.44 mm showing TdT^+^ cells in the entorhinal, perirhinal, and ectorhinal cortices of kindled (top panel) and control mice (bottom panel). Merged images show TdT^+^ in red and ChAT‐labeled cells in green. (B) Representative images in sagittal view at lateral 3.25 mm showing TdT activation in the entorhinal cortex during a seizure (left panel) and in a control mouse (right panel). (C) A greater number of TdT‐labeled cells were present in the lateral entorhinal cortex during a behavioral grade 2 seizure compared with controls (control 56 ± 7, seizure 145 ± 16; n = 5 mice per group, *t*(5.58) = 4.74, *P* = .004). (D) Similarly, more TdT^+^ cells were activated in the medial entorhinal cortex of mice with a grade 2 seizure (control 72 ± 10, seizure 117 ± 17; n = 5 mice per group, *t*(6.50) = 5.09, *P* = .002). Regions marked are CEnt, caudomedial entorhinal cortex; DLEnt, dorsolateral entorhinal cortex; Ect, ectorhinal cortex; MEnt, medial entorhinal cortex; PRh, perirhinal cortex; PrS, presubiculum, PaS, parasubiculum; Str, subiculum transistion area; S, subiculum

The parahippocampal region provides the primary cortical input to the hippocampus and encompasses the perirhinal, postrhinal, entorhinal cortices, and pre‐ and parasubiculum. Here, we observed that behavioral grade 2 seizures activated neurons in most of these areas. Within the EC, which provides direct cortical inputs into the hippocampus, there was a marked difference in neuronal activation between the lateral (LEC) and medial (MEC) cortices. We observed that the MEC, which interacts with the pre‐ and parasubiculum, postrhinal, and retrosplenial cortex, was more activated than the LEC connected to the perirhinal cortex, insular, and medial orbitofrontal, and olfactory areas, the caudomedial area of the MEC showing the most labeled cells (Figure 2). Additionally, the pre‐and parasubiculum was also labeled with TdT^+^ neurons. We observed TRAPed neurons in the perirhinal areas, which provide the primary neocortical input to the hippocampus directly and indirectly via the EC.

### 3.2 Cortical activation

**FIGURE 3:**
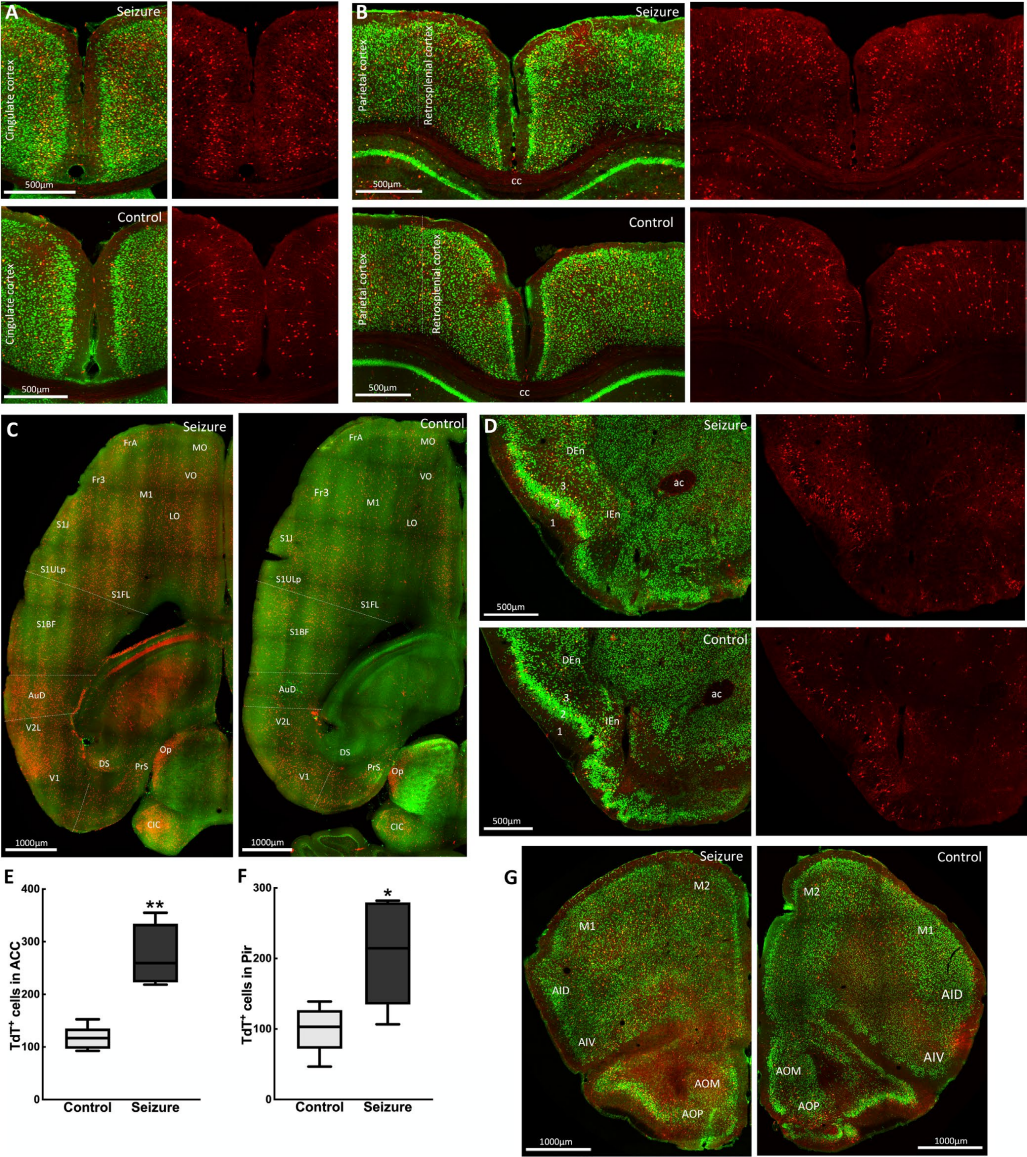
Multiple cortical structures are activated during a focal impaired awareness seizure. (A) Coronal sections at bregma 0.13 mm showing TRAPed neurons in the anterior cingulate cortex during a seizure (top panel) and in controls (bottom panel) in merged images showing NeuN immunolabels in green and TdT^+^ cells in red. (B) Coronal sections at bregma −1.91 mm showing TRAPed neurons in the retrosplenial and parietal cortices during a seizure (top panel) and in controls (bottom panel). (C) Horizontal sections at bregma −1.68 mm showing TdT activation in the frontal, orbital, motor, somatosensory, and visual cortices with (left panel) or without (right panel) a seizure. Merged images show TdT^+^ cells (red) and ChAT immunolabeling (green). (D) Coronal sections at bregma 1.09 mm showing TRAPed neurons in the piriform (layers 1, 2, and 3) and endopiriform cortex. (E) A greater number of TdT‐labeled cells were present in the ACC during a behavioral grade 2 seizure compared with controls (control 166 ± 10, seizure 274 ± 26; n = 5 mice per group, *t(*5.22) = 5.65, *P* = .002). (F) More piriform cortex cell body layer II neurons were TdT‐labeled compared with controls (control 100 ± 15, seizure 208 ± 33; n = 5 mice per group, *t*(5.56) = 2.91, *P* = .028). (G) Coronal sections at bregma 2.09 mm showing TRAPed neurons in motor and insular cortices, and olfactory nucleus. Regions marked are ac, anterior commissure; ACC, anterior cingulate cortex; AI, agranular insular cortex; AO, anterior olfactory nucleus; AuD, secondary auditory cortex; CIC, central nucleus of inferior colliculus; DEn, dorsal endopiriform nucleus; Fr, frontal cortex; IEn, intermediate endopiriform nucleus; M1, primary motor cortex; M2, secondary motor cortex; MO, medial orbital cortex; Op, optic nerve layer of superior colliculus; S1, primary somatosensory cortex; V1, primary visual cortex; V2, secondary visual cortex

The recruitment of cortical structures by a focal hippocampal seizure supports the manifestation of clinical symptoms. Following a seizure, we observed a bilateral activation of the left and right cortices, with the commissural fibers of the corpus callosum showing TdT‐labeling. TRAPed neurons were prominent in the cingulate cortex (Figure 3A), the retrosplenial cortex, the caudal portion of the cingulate cortex, and the parietal association cortex (Figure 3B). All areas of the motor, somatosensory, and primary and secondary visual cortices contained TRAPed neurons. TdT‐labeling was present in areas with high connectivity to the parahippocampal cortex, including the medial and dorsolateral prefrontal cortex and orbitofrontal cortex (Figure 3C). The piriform cortex, a part of the olfactory cortex, is interconnected with the olfactory bulb, hippocampus, and entorhinal cortex. Although there was sparse TdT‐labeling in the superficial plexiform layer I of the piriform cortex, a greater number of TRAPed neurons were present in the cell body layer II and in the endopiriform (layer IV) nucleus (Figure 3D). TRAPed neurons were present in the insular cortex, which mediates various sensory functions, including aversive states via its relay of external and interoceptive information. Within the anterior olfactory area, the medial and posterior subdivisions similarly contained greater numbers of TdT^+^ cells compared with the central part (Figure 3G).

### 3.3 Amygdala and associated structures

**FIGURE 4:**
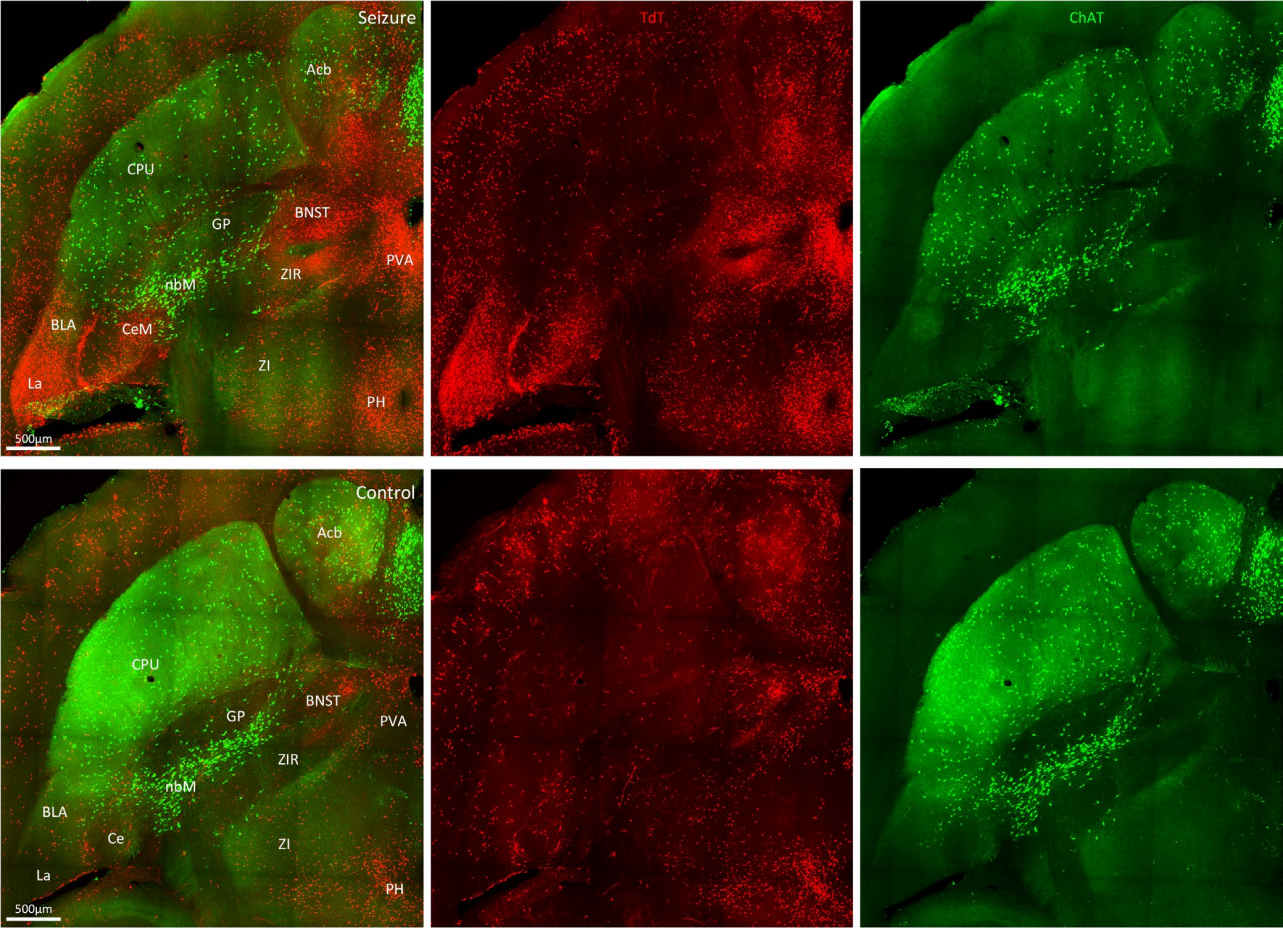
Activation in the amygdala and associated structures. Horizontal sections at bregma −4.28 mm showing TdT‐labeled cells (red) and ChAT‐labeled cells (green) and merged images of a mouse with a grade 2 behavioral seizure (top panel) and a control mouse (bottom panel). Regions marked are Acb, nucleus accumbens; BLA, basolateral amygdaloid nucleus; BNST, bed nucleus of the stria terminalis; CeM, central amygdaloid nucleus; CPu, striatum; GP, globus pallidus; La, lateral amygdaloid nucleus; nbM, nucleus basalis of Meynert; PH, posterior hypothalamic nucleus

Amygdala damage occurs in many TLE patients.^12^ The epileptic focus in TLE can reside in the amygdala. The amygdala receives sensory inputs from the cingulate cortex and visceral inputs from areas such as the septum, hypothalamus, and orbital cortex. It has a role in emotional behavior and cognitive function. In this study, we observed that a focal hippocampal seizure‐activated TdT^+^ cells of basal, central, lateral nuclei in this region (Figure 4). The lateral amygdala, an output nucleus of the amygdala with extensive projections to the temporal neocortex and hippocampus, was most prominently activated. Within the central amygdala, the medial amygdaloid nucleus had a greater number of TdT^+^ neurons. The bed nucleus of the stria terminalis (BNST) was also intensely activated with TdT. This region serves as a relay center between limbic structures, has reciprocal connections with the centromedial amygdala, and receives projections from the hippocampus and medial prefrontal cortex. Although the central amygdala and lateral BNST exhibit similar connectivity with other brain regions, the central amygdala receives cortical and sensory inputs while the BNST receives interoceptive feedback input from motor behavior systems and viscerosensory nuclei.^13^ Thus, it is involved in the control of autonomic, neuroendocrine, and behavioral responses.

We observed fewer cholinergic ChAT‐positive neurons of the nucleus basalis of Meynert in mice with a behavioral grade 2 seizure than unstimulated control mice (Figure 4). The nucleus basalis of Meynert is the principal source of cholinergic inputs to the cortex, olfactory tubercle, and amygdala. It shares bidirectional monosynaptic connections with sensorimotor areas involved in controlling attention and maintenance of arousal. Our findings are consistent with the reports that focal seizures result in decreased activity in this region in rodent models and human TLE patients.^14^, ^15^

### 3.4 Activation in the septal region

**FIGURE 5:**
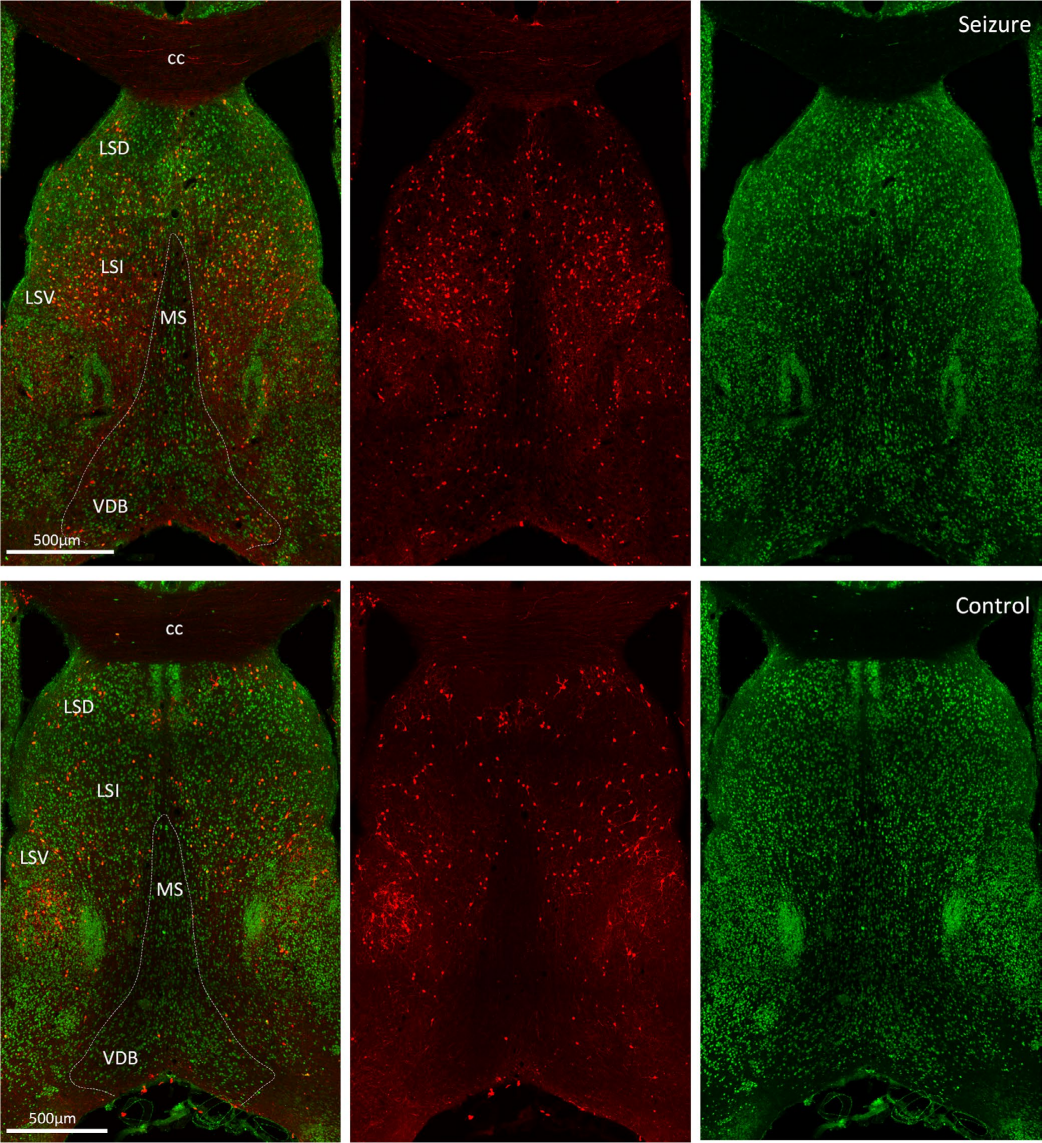
Neuronal activation in the septum. Coronal sections at bregma 0.97 mm showing activated TRAPed neurons in the septal area of a mouse with a grade 2 seizure (top panel) and an unstimulated mouse (bottom panel). Merged images show TdT^+^ cells in red and NeuN‐labeled neurons in green. Regions marked are cc, corpus callosum; LSD, dorsal lateral septum; LSI, intermediate lateral septum; LSV, ventral lateral septum; MS, medial septal nucleus; VDB, nucleus of vertical limb of the diagonal band

The septal nuclei share extensive reciprocal neural connections with the hippocampus, thalamus, hypothalamus, amygdala, habenula, and cingulate gyrus. TdT‐labeling was almost exclusive to the lateral septum with negligible medial septal activation (Figure 5). Within the lateral septum, activation was present in the caudal, rostral, and ventral subdivisions. Septo‐hippocampal fibers of the fimbria‐fornix also expressed TdT fluorescence. An alternate pathway, other than the fornix, for hippocampal projections to the anterior thalamic nuclei is through the internal capsule,^16^ which sits between the caudate nucleus and thalamus. In this structure, many projection fibers expressed TdT fluorescence.

### 3.5 Thalamic and subthalamic area activation

**FIGURE 6:**
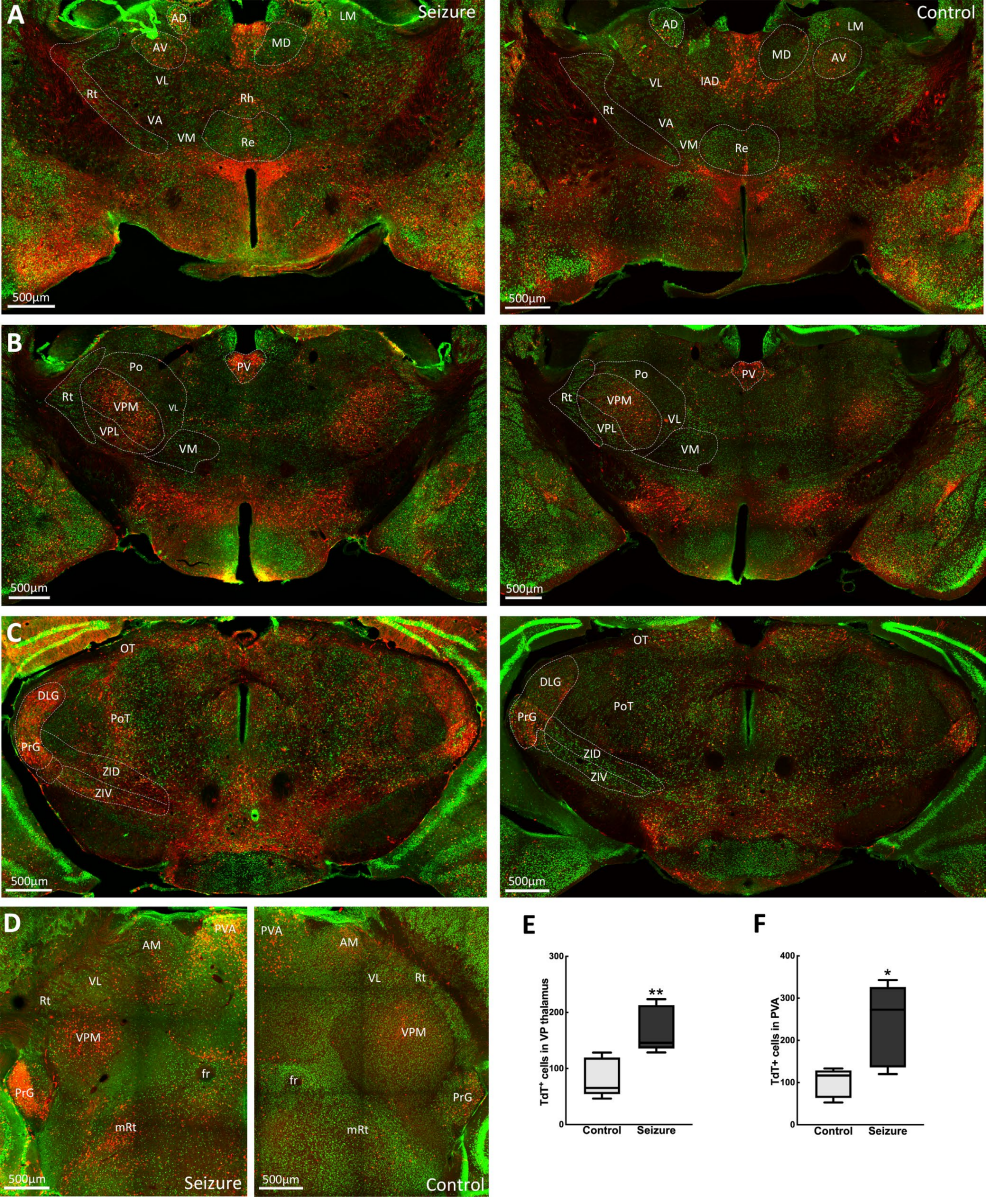
Activation of thalamic nuclei. (A–C) Coronal sections at bregma −0.95 mm, −1.43 mm, and −2.79 mm, respectively, show activation of multiple thalamic nuclei by a grade 2 seizure. (D) Images in horizontal view at bregma −3.28 mm showing activated TdT^+^ cells. Merged images show TdT‐labeled cells in red and NeuN‐labeled neurons in green. Regions marked are AD, anterodorsal; AV, anteroventral; DLG, dorsal lateral geniculate; fr, fasciculus retroflexus; MD, mediodorsal; mRt, mesencephalic reticular formation; OT, nucleus of optic tract; PrG, pregeniculate; Po, posterior thalamic nucleus; Re, reuniens thalamic nucleus; Rh, rhomboid; Rt, reticular nucleus; VA, ventral anterior; VL, ventrolateral; VM, ventromedial; ZI, zona incerta (E) TdT^+^ cell activation was greater in the PVA during a seizure (control 82 ± 15, seizure 168 ± 18; n = 5 mice per group, *t*(4.99) = 2.98, *P* = .031). (F) More thalamic VP neurons were TdT‐labeled compared with controls (control 100 ± 15, seizure 239 ± 44; n = 5 mice per group, *t*(7.76) = 3.54, *P* = .08)

Dense, direct projections to and from the subiculum and parahippocampal areas innervate the thalamus. Following a behavioral grade 2 seizure, there were TdT‐labeled neurons in sensory thalamic nuclei of the reticular and ventroposterior (VP) areas. However, the reticular nucleus with intrathalamic efferents was less active than the VP nuclei, which has reciprocal connections with the somatosensory cortex (Figure 6). We observed active neurons in the ventral posterolateral (VPL) nucleus, which receives nociceptive information via the spinothalamic tract, and the ventral posteromedial nucleus that receives sensory information from the face via the trigeminal nerve. In contrast, very few activated neurons were present in the motor ventral anterior, ventrolateral and ventromedial thalamic areas (Figure 6A).

Similarly, the posterior thalamic nuclei and regions of the anterior thalamus had sparse labeling with few TRAPed neurons. Within the lateral thalamus, the lateral geniculate nucleus, which relays visual sensory information, and the medial geniculate nucleus involved in the relay of auditory sensory information were activated. TRAPed neurons were also present among the midline thalamic nuclei, which send efferents to the hippocampal formation. Here, we observed activity in the paraventricular, mediodorsal, intermediodorsal, and central medial nuclei (Figure 6A,B). The TRAPed neurons were present in the zona incerta and subthalamic nucleus (Figure 6C). These subthalamic areas have extensive connections with multiple brain areas and are engaged in sensory motor activities.

## 4 DISCUSSION

We provide a comprehensive map of neuronal activation during a focal hippocampal seizure. We show significantly enhanced bilateral TdT activation in multiple hippocampal, cortical, and thalamic structures during behavioral grade 2 seizures, which contrasts with previous studies showing limited c‐Fos immunoreactivity during early seizure stages.^17^, ^18^, ^19^, ^20^, ^21^ For instance, our results indicate that during a focal hippocampal seizure, c‐Fos immunoreactivity is not limited to the bilateral hippocampi as previously shown in dorsal hippocampal kindled animals.^20^ Furthermore, we show that c‐Fos activity is also prominently increased in the dentate gyrus during grade 2 seizures, and not only during tonic‐clonic/grade 5 seizures as previously indicated.^21^

A key hypothesis in epilepsy is the dentate gate theory, where the hippocampal DG functionally gates the hippocampus from neuronal hyperexcitation, with a breakdown of gating implicated in seizure activity.^22^, ^23^ The normal hippocampus has a sparse number of activated DGCs with projections targeting the CA3 stratum lucidum, which also aids cognitive function and behavior.^24^ In contrast, we observed an extensive number of activated TdT^+^ DGCs during behavioral grade 2 seizures, consistent with a disruption of the dentate gate. Furthermore, these DGCs sent ectopic projections to both the CA3 stratum lucidum and stratum oriens, a phenomenon associated with postnatal development.^25^ This may either result from mossy fiber sprouting from mature DGCs or an increase in the number of newborn DGCs, but not the appropriate synaptic pruning that facilitates maturation of the hippocampal DG‐CA3 network to promote memory formation.^26^

Interactions within the hippocampus‐medial temporal lobe support memory consolidation. Disruption of these interactions as epileptiform discharges propagate through the same pathway may lead to impaired memory, as observed in some animal models and patients with TLE.^27^ Congruent with this, multiple structures in this system were activated during a focal seizure. CA3‐CA1 pyramidal neurons, which participate in spatial and episodic memory formation, were prominently active during seizures. This aligns with reports that hippocampal network desynchronization may underlie memory deficits in the epileptic brain.^28^ In the CA1‐subiculum‐EC relay pathway, the subiculum promotes seizure activity through increased glutamatergic signaling^29^ and inducts the EC into this activity. Under pathologic conditions, this pathway supports excessive propagation and loop‐gain amplification, which is a hallmark of TLE.^30^ Hippocampal epileptic discharges can cause the lateral septum to transition into an ictal state, which can be transmitted to the medial septum. More TdT^+^ cells were present in the intermediate lateral septum compared with other septal areas, which may align with reports that while most lateral septal neurons fired at a reduced rate compared with controls, a subpopulation transiently increased their firing around the interictal discharges in the chronic epilepsy model.^31^ During hippocampal kindling, medial septal cholinergic neurons produce an antiepileptogenic effect^32^ and could account for the lesser TdT activation observed in this area.

We also observed activation of multiple amygdaloid nuclei that modulate affectively‐influenced memory by regulating consolidation in other brain regions. Consequently, TLE patients with amygdala damage experience prolonged postictal confusion.^33^ In addition, TdT activation was enhanced in many cortical regions involved in memory formation, including the cingulate, retrosplenial, and piriform cortices. These cortices are extensively interconnected with other epileptogenic regions such as the hippocampus, rhinal cortex, and anterior thalamus. Thus, they provide a relay pathway for cortico‐cortical and cortico‐thalamic information, which could create a feedback loop for bilateral seizure spread.^34^

Based on our findings, if the memory pathway serves as a potential route for the propagation of seizure activity, this could account for memory difficulties reported in some TLE patients. Consequently, some TLE patients with a hippocampal focus experience episodic memory deficits.^35^, ^36^ Patients with a left‐sided focus also appear to have markedly more memory problems.^37^

Structural and functional changes in frontal limbic structures such as the hippocampus, amygdala, and medial prefrontal cortex are implicated in some psychiatric disorders, which are common comorbidities in patients with TLE. Anxiety and depression are associated with altered glucocorticoid secretion due to a dysfunctional hypothalamic‐pituitary‐adrenal (HPA) axis, and HPA axis hyperactivity is evident in TLE patients even after prolonged interictal periods.^38^ HPA axis regulation by the limbic structures is region‐ and stressor‐specific and occurs via intermediary neurons in the BNST, hypothalamus, and brainstem.^39^ BNST GABAergic neurons, which we observed to be prominently labeled during a grade 2 seizure, integrate and relay physiological and behavioral responses between these limbic structures.

A relationship exists between increasing amygdala volume and the severity of depression symptoms.^40^ The amygdala activates the HPA axis via the medial and central amygdaloid nuclei, which were active during a grade 2 seizure. These nuclei are a primary target of cortico‐sensory systems, producing autonomic responses such as changes in heart rate, blood pressure, and respiration that occur during temporal lobe seizures. In addition, the lateral amygdala, which mediates associative processing of both neutral conditioned and aversive unconditioned stimuli,^41^ was intensely activated. These amygdaloid nuclei are known to show stress‐induced Fos activation.^42^ The ventral hippocampus supports emotional and affective processes.^43^ This region contains many glucocorticoid receptors, which facilitate detection of glucocorticoid concentrations and mediate feedback inhibition of the HPA axis via CA1 and ventral subiculum projections to the hypothalamus. Similarly, neurons of the anterior cingulate of the medial prefrontal cortex activated during grade 2 seizures in this study are implicated in HPA axis inhibition and show c‐Fos activation in response to stressful stimuli.^42^ Thus, chronic seizures may induce cellular network reorganization, which alters HPA axis function to produce depressive symptoms.

Seizure generalization may occur from limbic structures via the thalamus and other subcortical structures.^44^ Thalamic areas that share connections with the somatosensory cortex, including the paraventricular and VP thalamus, were more activated by a seizure compared with unstimulated mice. VP relay nuclei thalamocortical oscillations have also been implicated in generalized absence seizures, which present with similar clinical symptoms as a focal impaired awareness seizure. The zona incerta is associated with major motor control tracts and may support the control of stereotyped automatisms,^45^ such as observed during focal impaired awareness seizures. Notably, zona incerta and VPL thalamus are sites for deep brain stimulation (DBS) in diseases such as Parkinson's and essential tremor,^46^, ^47^ so might be potential DBS targets in intractable TLE, where current targets include the hippocampus and anterior and centromedian thalamus.^48^

We have shown brain‐wide mesoscale neuronal activation during a focal impaired awareness seizure in electrically‐kindled mice. The circuits mapped in this study are implicated in TLE as well as associated comorbidities such as memory deficits, depression, and anxiety disorders. The possible hijack of memory and stress systems by a focal impaired awareness seizure could account for the high vulnerability of persons with complex partial seizures to these comorbidities. Kindling is a reliable model of TLE, which produces a series of reproducible cellular and molecular alterations in the neural circuitry relevant to epileptogenesis.^49^ However, this procedure is limited by failure to produce chronic spontaneous seizures and some histopathologic changes associated with TLE. Although using TRAP mice with permanent IEG tags provides a better spatial resolution of neuronal activity during seizures, we cannot conclusively outline the propagation pattern of these seizures. Another caveat is that we did not assess IEG expression or test behaviors during interictal periods. Further studies to regulate the activity of specific cells to link neuronal activation patterns to specific seizure behavior will enhance our knowledge of these parameters.

## CONFLICT OF INTERESTS

None of the authors has any conflict of interest to disclose. We confirm that we have read the Journal's position on issues involved in ethical publication and affirm that this report is consistent with those guidelines.
